# Transgenic expression of flavanone 3‐hydroxylase redirects flavonoid biosynthesis and alleviates anthracnose susceptibility in sorghum

**DOI:** 10.1111/pbi.13397

**Published:** 2020-06-17

**Authors:** Lanxiang Wang, Andy C.W. Lui, Pui Ying Lam, Guoquan Liu, Ian D. Godwin, Clive Lo

**Affiliations:** ^1^ School of Biological Sciences The University of Hong Kong Hong Kong China; ^2^ Research Institute for Sustainable Humanosphere Kyoto University Kyoto Japan; ^3^ Centre for Crop Science Queensland Alliance for Agriculture and Food Innovation The University of Queensland Brisbane QLD Australia

**Keywords:** sorghum, flavanone 3‐hydroxylase, 3‐hydroxylated flavonoids, anthracnose

Flavonoids are ubiquitous in terrestrial plants with important physiological functions. The *in planta* flavonoid profile depends on the activities of different biosynthesis enzymes (Figure 1a). Flavanone 3‐hydroxylase (F3H) is a key enzyme channelling carbon flow towards the production of 3‐hydroxylated flavonoids, including flavonols and anthocyanidins. In Poaceae, F3H‐encoding genes are generally inactive in vegetative tissues which accumulate flavone derivatives as the predominant flavonoid metabolites. Meanwhile, sorghum produces 3‐deoxyanthocyanidins and flavones as phytoalexins for defence against pathogens such as *Colletotrichum sublineola*, the causal agent of anthracnose. The occurrences of 3‐hydroxylated flavonoids in sorghum are generally not well known. Only in some cultivars, anthocyanin pigments accumulate in mesocotyls of seedlings upon illumination following the activation of *F3H* and other anthocyanidin biosynthesis genes.

**Figure 1:**
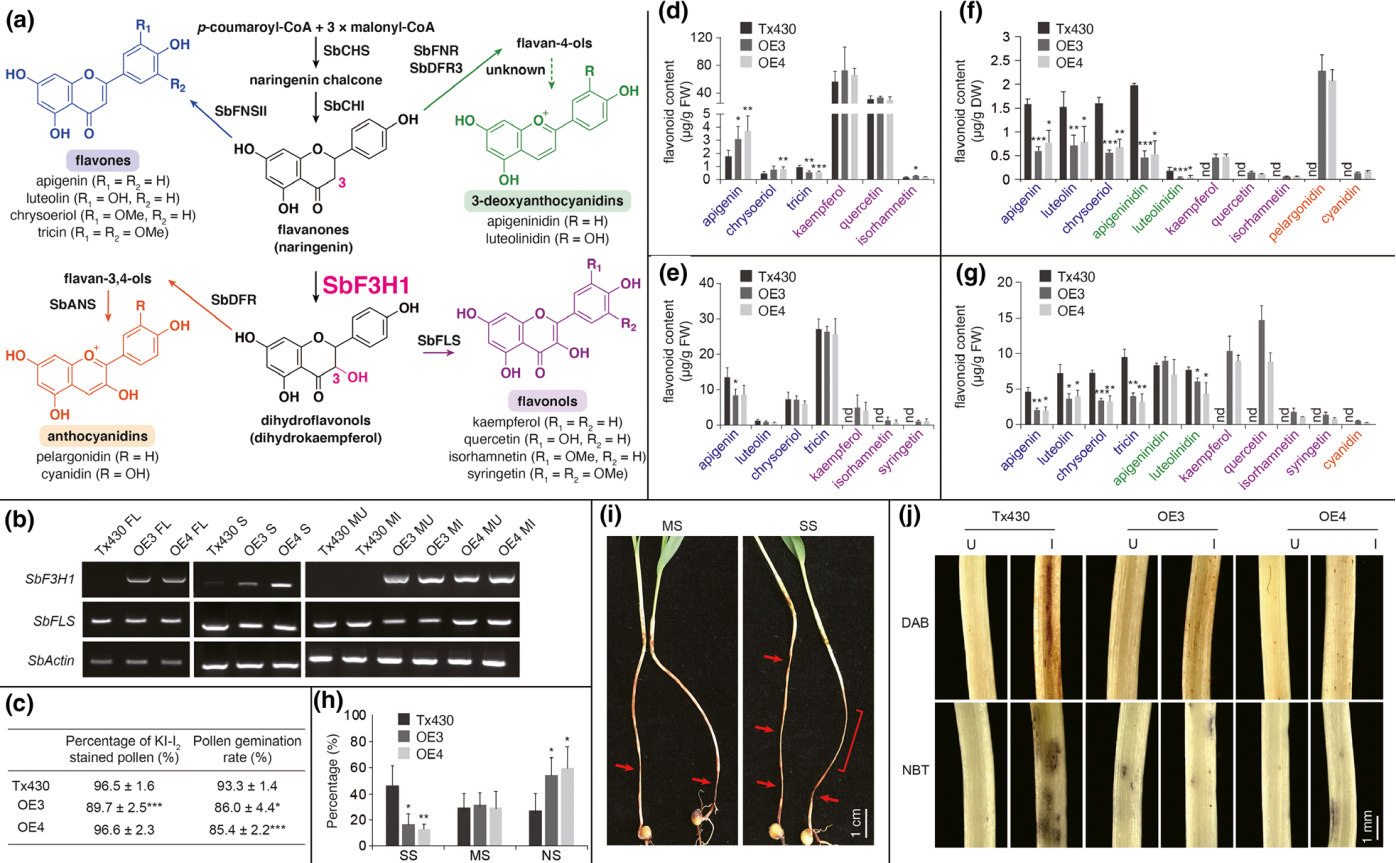
*SbF3H1* overexpression in sorghum. (a) Flavonoid biosynthesis pathway in sorghum; (b) RT‐PCR expression analysis of *SbF3H1* and *SbFLS1* in different tissues of Tx430 control and *SbF3H1*‐overexpressing lines (OE3 and OE4); (c) Pollen viability and pollen germination analyses; (d–g) Accumulation of different flavonoids in (d) spikelets (before anthesis), (e) flag leaves (booting stage), (f) seeds and (g) mesocotyls 72 h after inoculation with *C. sublineola*; (h) Disease severity of mesocotyls 6 days post‐inoculation; (i) Examples of mild symptom (MS) and strong symptom (SS); Areas of lesion are indicated in red; Scale bar = 1cm. (j) ROS straining of mesocotyls 24 h post‐inoculation; Scale bar = 1mm. ANS, anthocyanidin synthase; CHS, chalcone synthase; CHI, chalcone isomerase; FNR, flavanone 4‐reductase; DFR, dihydroflavonol 4‐reductase; F3H, flavanone 3‐hydroxylase; FNS, flavone synthase; FLS, flavonol synthase; FL, flag leaf; S, spikelets; M, mesocotyls; FW, fresh weight; DW, dry weight. nd, not detected; I, inoculated; U, uninoculated; NS, no symptom. Data represent mean ± SD (*n* = 3). *P < 0.05, **P < 0.01, ***P < 0.001 (Student’s *t*‐test).

Here, we transformed the sorghum inbred line Tx430 with the coding region of *SbF3H1* (Sb06g031790) under the control of a maize *ubiquitin1* (*ubi1*) promoter for flavonoid profiling and disease phenotyping. Two independent transformants (OE3 and OE4) were selected for characterizations in T_3_ generation. *SbF3H1* expression was detected in different tissues of the OE lines, but only in spikelets of Tx430 control (Figure 1b). Both OE lines showed no observable phenotypes from seedling to ripening stages. However, seed numbers per head declined by 55.6% and 18.8% in OE3 and OE4, respectively, when compared to Tx430 control. This is likely due to subtle pollen defects; slight reductions in percentage of viable pollen and pollen germination rate were found in OE3 and both lines, respectively (Figure 1c). In contrast, the grain yield component of 100‐seed weight showed a 10% increase in OE3 but remained unchanged in OE4.

Acid‐hydrolysed extracts of different tissues were prepared for LC‐MS/MS analysis. The flavonols kaempferol, quercetin and isorhamnetin were detected in all spikelet extracts analysed, while isorhamnetin accumulated in slightly higher amount in OE3 than Tx430 control (Figure 1d). Hence, overexpressing *SbF3H1* did not have a substantial impact on flavonol production in tissues with endogenous F3H activities. The presence of high levels of flavonol derivatives in spikelets of wild‐type sorghum suggests a potentially important role for male fertility as demonstrated in other plant species. For instance, flavonols function as antioxidants to regulate reactive oxygen species (ROS) homeostasis during pollen tube elongation in tomato (Muhlemann *et al.*, 2018).

In flag leaf extracts, kaempferol, isorhamnetin and syringetin were identified in both transgenic lines, but none of these flavonols were found in Tx430 (Figure 1e). It is worth‐noting that syringetin (3′,5′‐*O*‐dimethylated flavonol) is absent from flavonol‐producing spikelets of Tx430 control. Formation of syringetin requires flavonoid 3′,5′‐hydroxylase (F3′5′H) activities to generate 3′,5′‐hydroxyl groups for *O*‐methylation. In some dicots, F3′5′Hs belonging to the P450 CYP75A subfamily are responsible for 5′‐hydroxylation of different flavonoid substrates (Seitz *et al* *.*, 2006). Interestingly, a chrysoeriol‐specific 5′‐hydroxylase belongs to CYP75B subfamily is involved in tricin (a 3′,5′‐*O*‐dimethylated flavone) biosynthesis in grasses (Lam *et al.*, 2019). Whether a CYP75A F3′5′H or a CYP75B flavonol‐specific 5′‐hydroxylase is required for syringetin biosynthesis in leaf tissues of transgenic *SbF3H1* lines remains to be elucidated.

Flavonol synthase (FLS) is a downstream enzyme of F3H during flavonol biosynthesis. The first monocot FLS‐encoding gene functionally characterized was maize *ZmFLS1* with a tandemly duplicated gene *ZmFLS2* (Falcone Ferreyra *et al.*, 2012). Both genes are constitutively expressed in most maize tissues and can be further induced by UV‐B treatment. Sorghum SbFLS1 is a highly conserved homolog of ZmFLSs (>96% sequence identity). Similarly, *SbFLS1* is expressed in all tissues examined in Tx430 control (Figure 1b), suggesting that *SbF3H1* expression may modulate flavonol production. Hence, flavonol derivatives were detected in spikelet but not in other tissues which lack *SbF3H1* expression in wild‐type sorghum.

Flavone derivatives are the prevalent flavonoid metabolites in grass biomass. Consistently, apigenin, chrysoeriol and tricin were detected in spikelet samples, while apigenin and tricin were slightly increased and reduced in the OE lines, respectively (Figure 1d). Meanwhile, apigenin, luteolin, chrysoeriol and tricin were found in flag leaf samples. Compared with Tx430 control, level of apigenin was reduced in OE3 (Figure 1e).

Sorghum bran contains substantial amounts of 3‐deoxyanthocyanidins (Awika *et al.*, 2004). Consistently, apigeninidin and luteolinidin were detected in Tx430 control whole seed extracts (Figure 1f). Interestingly, anthocyanidins (pelargonidin and cyanidin), besides flavonols, were detected in seed of both transgenic lines as 3‐hydroxylated flavonoids. Anthocyanidins and 3‐deoxyanthocyanidins are structurally similar except for the C3‐hydroxylation. As the endogenous 3‐deoxyanthocyanidin branch pathway is active in seeds, some enzymes may accept 3‐hydroxylated substrates for anthocyanidin biosynthesis. For example, two dihydroflavonol reductases (SbDFR1 and SbDFR3) could reduce dihydroflavonols and flavanones to produce the immediate precursors of anthocyanidins and 3‐deoxyanthocyanidins, respectively (Liu *et al.*, 2010).

The elongated mesocotyl system was further used to investigate flavonoid profiles and disease phenotypes in sorghum seedlings following *C. sublineola* infection. LC‐MS/MS analysis revealed the accumulation of flavonol derivatives and cyanidin in inoculated mesocotyls of transgenic seedlings but not in those of Tx430 (Figure 1g). Meanwhile, accumulation of 3‐deoxyflavonoid phytoalexins (3‐deoxyanthocyainidin and flavones) was reduced in transgenic seedlings compared with Tx430 seedlings. Although flavonoid biosynthesis is activated considerably after infection (primarily through chalcone synthase induction), SbF3H1 is apparently diverting some of the carbon flow towards 3‐hydroxylated flavonoid production.

Expression of flavone biosynthesis genes [flavone synthase (*SbFNSII*; Sb02g000220), apigenin 3ʹ‐hydroxylase/chrysoeriol 5ʹ‐hydroxylase (*SbA3ʹH/C5ʹH*; Sb09g022480) and flavonoid *O*‐methyltransferase (*SbFOMT*; Sb07g003860)] was examined in spikelets, flag leaf and infected mesocotyls. No significant changes in gene expression levels were found between Tx430 and both transgenic lines, except a slight increase in the expression of *SbFOMT* was detected in flag leaf of OE3 (data not shown). Meanwhile, expression of 3‐deoxyanthocanidin biosynthesis genes [flavanone 4‐reductase (*SbFNR*; Sb06g029550) and *SbDFR3* (Sb04g004290)] was not altered in infected mesocotyls of the OE lines. These results suggest that overexpressing *SbF3H1* did not result in major alterations in expressions of genes in other flavonoid branch pathways.

For inoculated mesocotyls, disease severity was examined 6 days post‐inoculation as described (Schnippenkoetter *et al.*, 2017). Occurrences of strong symptoms in Tx430 mesocotyls were 64% more compared with both transgenic mesocotyls (Figure 1h,i). By contrast, there were more transgenic seedlings with no disease symptoms than Tx430 control. Hence, *SbF3H1* overexpression resulted in reduced susceptibility to *C*. *sublineola* albeit lower amounts of 3‐deoxyflavonoid phytoalexins were produced. Because sorghum pathogens are less likely to encounter flavonol and cyanidin derivatives in vegetative tissues of wild‐type sorghum, there may not be existing detoxification mechanisms in the fungus. In addition, these 3‐hydroxylated flavonoids may function synergistically with 3‐deoxyflavonoid phytoalexins to improve the overall antifungal capacity in the *SbF3H1*‐overexpressing plants.

Hydrogen peroxide and superoxide ion were analysed in sorghum mesocotyls by diaminobenzidine (DAB) and nitro blue tetrazolium (NBT) staining, respectively (Figure 1j). In uninoculated seedlings, there was little or no DAB and NBT staining. Brownish DAB lesions were evident in Tx430 control mesocotyls 24 h post‐inoculation but barely observable in the transgenic mesocotyls. Similarly, NBT staining was more intense and widespread in infected Tx430 seedlings compared with infected transgenic seedlings. Overall, pathogen‐inducible ROS production is alleviated in the *SbF3H1*‐overexpressing lines. As flavonol derivatives are potent antioxidants (Heim *et al.*, 2002), they may scavenge some ROS in the infected transgenic seedlings.

Previously, transgenic *F3H* overexpression was performed in tobacco which naturally accumulates 3‐hydroxylated flavonoids due to endogenous F3H activities, leading to enhanced accumulation of flavonols and flavan‐3‐ols without vigorous changes in flavonoid profile (Song *et al.*, 2016). By contrast, we overexpressed *SbF3H1* in sorghum which is deficient in 3‐hydroxylated flavonoids in vegetative tissues, resulting in partial re‐direction of carbon flow towards 3‐hydroxylated flavonoid production, hence increasing the complexity of flavonoid‐derived metabolites in different tissues. The enriched flavonoid profile may enhance defence response and improve nutraceutical values of sorghum grain/bran. In particular, syringetin derivatives, which are novel flavonoid metabolites not identified in sorghum previously, showed a range of health beneficial properties (Hsu *et al.*, 2009). Considering seed yield reduction in the overexpression lines, the use of pathogen‐inducible and tissue‐specific promoters should be explored for driving targeted *SbF3H1* expression without compromising grain productivity.

## Conflicts of interest

The authors declare that there is no conflict of interest.

## Author contributions

LW, IDG and CL designed the experiments. LW, ACWL and GL performed the experiments. LW, ACWL, PYL, IDG and CL analysed the data. LW, ACWL, PYL and CL wrote the manuscript with contributions of all other authors.
